# Atypical Black Leader Emergence: South African Self-Perceptions

**DOI:** 10.3389/fpsyg.2021.626473

**Published:** 2021-03-08

**Authors:** Angel Myeza, Kurt April

**Affiliations:** ^1^Graduate School of Business, University of Cape Town, Cape Town, South Africa; ^2^Allan Gray Centre for Values-Based Leadership, Graduate School of Business, University of Cape Town, Cape Town, South Africa

**Keywords:** self-perceptions, atypical, leadership, psychological triggers, colorism, ubuntu, generational trauma, black – African

## Abstract

The research aimed to gain understanding of the self-perceptions of black professionals in relation to business leadership, and how these self-perceptions influenced their behaviors, aspirations and self-perceived abilities in leadership positions. The study was specifically focused on black South African professionals. Black professionals were found to exhibit signs of deep-rooted pain, anger and general emotional fatigue stemming from workplace-, socio-economic- and political triggers that evoked generational trauma and overall negative black lived experiences. The negative lived experiences could have led to racial identity dissonance and, in extreme cases, complete racial identity disassociation. Moreover, black professionals were found to display symptoms of ‘survivor guilt,’ stemming from the shared history of oppression amongst black people in South Africa. The ‘survivor guilt’ contributed toward a profound sense of shared responsibility and purpose to change the circumstances, experiences and overall perceptions about the capabilities of black professionals. Results showed that upbringing, determination, resilience, black support networks, and black leadership representation within organizational structures were important ingredients that positively contributed to the leadership aspirations and success of black professionals. The research discovered that, in some cases, black professionals leveraged white relationships to propel their careers forward, however, this practice reportedly resulted in the black professionals experiencing feelings of self-doubt in their own abilities. Self-doubt, also found to be a result of historical oppression, could have and have been shown to eventually lead to self-deselection, negatively impacting the aspirations and career advancement prospects of black professionals in organizational leadership. Furthermore, the research found that black leaders believed that their blackness, specifically, its unique texture of experiences and history in South Africa, provided them with superior empathetic leadership abilities toward other black employees. Black leaders frequently highlighted the distinctive values of ubuntu as the cornerstone of their leadership approach. In addition, it was found that black professionals also considered their blackness, particularly the shade of their skin, to detract from their leadership opportunities, as it reduced the odds of being authorized as natural leaders, thus fortifying a skewed self-perception of their own leadership capabilities.

## Introduction

Our research aimed to gain understanding of the self-perceptions of atypical black professionals in relation to business leadership and how these self-perceptions influence the emergence, or lack thereof, in terms of their behaviors, aspirations, and self-perceived abilities in leadership positions prototypically held by white, mainly male, professionals. We argue that both political- and social power continue to fail to unseat the patterns of economic power and historic practices, embedded over centuries of oppression in South Africa, that ascribes white males to positions of business leadership. Samdanis and Özbilgin (2020) point out that organizational leadership is still dominated by select white males from privileged backgrounds, despite the impetus to get more individuals from atypical backgrounds into leadership roles. As a result, black professionals either push themselves forward through purposeful, sometimes unconventional pathways, or self-sanction themselves and hinder their own career progress, due to the assumptions and opinions they hold about their own and white men’s abilities.

Africa is a continent with a history of colonialism, which ingrained Western forms of knowledge and organizational structures while suppressing African identities, cultures, values and epistemologies (Nkomo, 2015). Even Africa’s knowledge about leadership is largely based on theories the West has provided, starting as far back as the colonial times till today (Theimann et al., 2006). Decolonization, which can be described as economic emancipation as well as liberation from Western knowledge domination, thus remains a priority for many countries in Africa (Nkomo, 2015). According to Ibekwe, 1987, the central theme of ‘decolonizing the mind’ is to overthrow the stronghold, even the authority, that colonizer traditions and belief systems have over Africans. This, in his view, demands a dismantling of the beliefs and assumptions we hold as “truths” on an individual level, as families, as communities and in our organizations, and we are encouraged to critically interrogate and critique the systems which uphold them. Decolonization does not mean ignorance of colonizing ideas, notions, traditions and structures of power – it simply means a denial of their authority over African thinking and ways of being, a moving away from blind allegiance to them and, in particular, in our notions of what, and which pathways, determine competent leadership.

Our research aimed to add to intersectional leadership knowledge from a context that is under-researched, i.e., interrogating what the leadership self-perceptions and associated behavioral paths of atypical black South African professionals, living and working in the economic hub of South Africa (Gauteng) and exposed to colonization, racism and apartheid, are. Mzileni (2017) notes that only ideas, practices and concerns produced by white institutions tend to be recognized, while Theimann et al. (2006) as well as Ospina and Foldy (2009), observed that African theories are rarely captured in global organizational- and leadership literature, thus emphasizing the importance of developing theory in the African context.

## Literature Review

We briefly draw on a number of theories that were relevant to our topic under discussion.

### Atypical Leaders

An atypical leader is described as an individual that is *‘rarely associated with leadership positions,’* (Samdanis and Özbilgin, 2020, p. 101). Furthermore, according to Samdanis and Özbilgin (2020), an atypical leader comes from an unprivileged, minority, under-represented, deprived or an unusual demographic environment, example; people that come from inferior socio-economic circumstances. However, Samdanis and Özbilgin (2020) also specify that not all atypical leaders have the same degree of atypicality, instead, it varies based on the mix of their unique beliefs about their social identities. In South Africa, black professionals, and the subsequent leaders that emerge are atypical because, although black people are not a minority in the country, they, however, remain underrepresented and often, stereotyped as unsuitable for leadership roles, and they have come from unprivileged conditions and poor socio-economic conditions. As an example, Méndez-Morse (2000) explains why Latina women in leadership, specifically those that held superintendent positions, were deemed atypical: Firstly, that the Hispanic women were stereotyped regarding what they were and what they could be, secondly, that historically Latina leaders were not portrayed in leadership which strengthened the stereotype and, lastly, that research rarely included minority women in leadership. Similarly, black South African leaders are stereotyped as unfit for leadership, are stereotyped in terms of what they are and could be and are rarely portrayed in leadership, and just as rarely included in leadership research, thus further fortifying the belief that black South African leadership is atypical.

### The Emergence of Atypical Leaders

Acton et al. (2019, p. 8) define leadership emergence as *‘the multilevel interactional process driven by deep level cognitive and perceptual processes of group members that form a collective patterning of leader and follower interactions over time,’* while Kalish and Luria (2016) define an emergent leader as one who, despite not holding any official position of leadership, is perceived as a leader by others. An emergent leader has prominence and the ability to heavily influence a group (Kalish and Luria, 2016). Samdanis and Özbilgin (2020) assert that the emergence of an atypical leader should not be viewed as an exception in companies that are committed to transformation and diversity, instead, it should only be a challenge to organizations that do not practice diversity. We argue that, in South Africa, although organizations are held to account on diversity initiatives with most of them being highly committed to these initiatives, the rise of atypical leaders remains a steep challenge in these organizations.

Nkomo (2015) shares that during the apartheid era in South Africa, black people – African, Colored and Indian - were excluded from managerial and professional positions. In South Africa, the apartheid system created a racial hierarchy in the workplace that firmly placed the white man at the top (Nkomo, 2015). This privilege still prevails in modern society where white is often considered as the principal trait of leadership (Cranmer and Harris, 2015). According to Nkomo (2015), top and senior management positions are still dominated by white males.

Nkomo (2011) explains that colonization in Africa fostered the view that all things European were positive whilst forging the perception that all things African were negative. We argue that present day South African organizations still utilize these lenses to justify limiting leadership opportunities for black professionals and that there is a biased view to label them incompetent and unsuitable for leadership. Acton et al. (2019) state that in the process of leadership emergence, the prospective leader must possess the characteristics and behaviors of the perceivers’ leadership ‘prototype,’ such that there is a ‘match,’ before the prospective leader is granted the category of ‘leader’ to the respective group.

### Black Identity and Black Leadership

#### Black Identity

Pierre and Mahalik (2005) define positive racial identity as: *“*… *the process of development by which individual members of various socio-racial groups overcome the version of internalized racism that typifies their group in order to achieve a self-affirming and realistic racial-group or collective identity”* (p. 30). Lightfoot and Foster (1970) state that black identity has many stages of development and growth that reveal socio-cultural beliefs of that time and thus, identity is not fixed but evolves. Hall et al. (1972) suggest that there are four stages of black identity development, as observed in black Americans when they would discover blackness in themselves: (1) pre-encounter, (2) encounter, (3) immersion, and (4) internalization. Moreover, Hall et al. (1972) note that the result of this encounter with blackness is that the person will thereafter define themselves as being ‘black, adequate and non-inferior’ (p. 4).

Steve Biko, founder of the Black Consciousness movement in South Africa, positively identified ‘black’ with characteristics such as independence, self-reliance and assertiveness (Lloyd, 2003). Black Consciousness, developed in 1970, demanded that black South Africans rethink their identities (Heffernan, 2015), particularly in relation to taking up more powerful roles in society and in business. The term ‘black’ was hardly used before the Black Consciousness Movement adopted it in 1970 (Lloyd, 2003). Before this adoption, ‘non-white’ was the term used to describe people of a certain race by the apartheid government in South Africa (Lloyd, 2003) – signifying that the base reference for all people of color in South Africa was in relation to whiteness. Steve Biko felt that the term ‘non-white’ was an annulment of ‘being’ and felt that the term ‘black’ could instead be used as a positive term of affirmation (Lloyd, 2003). Paasche (2017) states that Africans can only regain their identity by claiming back what belongs to them and telling their own story themselves – which, in leadership literature and practice, is rarely done and thus the reproduction of the old leadership elite ensues, together with a dearth of stories about atypical, socio-relational pathways to leadership.

#### Black Leadership

Nkomo (2011) describes African management thought as one that is guided by traditional values and principles but explains that, broadly, African leadership has largely been portrayed as deficient, a view that is deeply rooted in the colonial beliefs about Africa. Furthermore, Nkomo (2011 p. 376) explains that Ubuntu - defined as *‘humaneness—a pervasive spirit of caring and community, harmony and hospitality, respect and responsiveness—that individuals and groups display for one another. Ubuntu is the foundation for the basic values that manifest themselves in the ways African people think and behave toward each other and everyone else they encounter’-* is an African philosophy, and is highly connected to leadership and management in Africa. Ubuntu is believed to have originated in South Africa (Nkomo, 2011; Showunmi et al., 2016), and can be viewed as a potential competitive advantage (Nkomo, 2011).

It is noted that perceptions of leadership, either by oneself or others, are dependent on context and can vary depending on race (Festekjian et al., 2014). The experiences and life histories of leaders of color make them more likely to vicariously and compassionately experience their subordinates suffering – modeling appropriate responses to others in the organization (Kanov et al., 2004). Leigh and Melwani (2019) claim that leaders’ compassion, and organizational climates of inclusion, serve to empower black employees through the noticing of distress in black employees and offering assistance (listening, counseling, and advocating). Additionally, because compassionate leaders are likely to display concern for their followers’ well-being (Scott et al., 2010), this should increase self-disclosure, setting the foundation for relational bridging ties. April et al. (2000) state that the 1994 promise of the new, democratic South African context opened the possibility for multiple, equally valid realities, held by all of its diverse groups from different cultures and different walks of life, with a leadership movement toward full participation, collaboration and enriched relationships. Sadly, Ospina and Foldy (2009) state that leaders of color are at a disadvantage because they tend to be viewed as less legitimate and, because of this, they may enact their leadership differently. Kuada (2010) suggests that leadership in Africa is shaped to a large extent by culture and historic events. There is an argument that research needs to delve deeper into black leadership – particularly how African leaders behave, why they behave in the ways that they do, and the impact of those behaviors on organizations (Kuada, 2010). Bartley (2013) suggests that certain dynamics, to some extent, exist within black leaders which she refers to as ‘internal defaults.’ Examples of these defaults are ‘not good enough,’ ‘silenced,’ ‘dis-guarded,’ ‘furtive,’ ‘black in the negative’ - all of these in addition to other external behaviors that black leaders are subjected to Bartley (2013). Nkomo (2011) suggests that Africa needs to seek its own solutions and approaches to leadership and management, to reclaim the identity of its people.

### Self-Perception Theory

Self-perception theory states that: *“Individuals come to ‘know’ their own attitudes, emotions, and other internal states partially by inferring them from observations of their own overt behavior and/or the circumstances in which this behavior occurs”* (Bem, 1972, p. 2; Fazio et al., 1977; Chaiken and Baldwin, 1981). Fazio et al. (1977) further explain that self-perception theory predicts that a new attitude shall occur if a person acts out a behavior that is more extreme than is inferred by his or her attitude. There are two main dimensions of self-perception which are positively interrelated – self-esteem and personal efficacy (Hughes and Demo, 1989). An important difference of self-perception theory to dissonance theory is that, in contrast, dissonance theory predicts attitude change only occurring if the behavior is in disagreement with the attitude (Fazio et al., 1977).

### Psychology – Intrapersonal Influence

Race is a huge part of how people view themselves and others and, as such, important in the role it plays in leadership perception (Festekjian et al., 2014). Psychological research shows that context can make an individual aware of the perceptions of leadership regarding his/her own race and that this can, in turn, negatively influence their own leadership ambitions (Festekjian et al., 2014). Nkomo (2015) points out that during the apartheid era in South Africa, the ambitions of Africans could only reach as far as aspiring to becoming ‘boss boys.’ In organizations that have initiatives such as employment equity and affirmative action (which are created to try and rectify issues of diversity), employees are frequently left feeling excluded, unaffirmed and undervalued in their own identity, making them believe that they are not deserving of promotions even if they would have previously believed themselves to be competent (April et al., 2012). Research shows that potential leaders may internalize leadership beliefs held by others (Festekjian et al., 2014). Lowe (2013) stated that the experience of projective identification, a process through which one group serves as a ‘sponge’ for all the negative feelings of another group, is deeper than is generally recognized because, through projective identification, many black people discount themselves as not being good enough to become leaders, and withdraw their motivations, especially as senior leaders within the organizations they work for.

Additionally, intrapersonal leadership perceptions are influenced by race and this, in turn, influences the individual’s leadership aspirations (Festekjian et al., 2014).

### Black Psychology

Even though some state that the science of behavior is universal, there is ample cross-cultural evidence that indicates that the data of psychology is, in actual fact, not universal (Underwood, 1972). Jamison (2018) provides a few definitions of black psychology, including: *“*… *the study of the behavioral patterns of black people in a social environment that is manifestly antagonistic and unhealthy*… *It is concerned with developing appropriate methodologies and tools required for valid analysis of the black experience”* (p. 725). Jamison (2018, p. 726) also cites the definition by Wilson (1998) which provides the following explanation*: “*… *it is a psychology of liberation.”* White (1970) states that traditional theories developed by white psychologists to describe white people, are not suitable to be utilized to try to comprehend the lifestyles of black people because, when these theories are applied to the lives of black people, it is very likely that incorrect deductions are drawn. Furthermore, in South Africa, Black Consciousness highlighted and was based on the basis that apartheid or race-based oppression had crippled the ways in which black people perceived themselves and their place in their birth country, to the extent that they needed psychological freedom before they could achieve political- and economic freedom (Heffernan, 2015).

Alleyne (2004) suggests that black people carry with them persistent ‘post-slavery traumatic stress syndrome’ and that there is a need to investigate its precise magnitude. She firmly believes that there is a ‘post-colonial, post-slavery’ context whose baggage is passed from one generation to the next (Alleyne, 2004). Leigh and Melwani (2019) suggests that, because of this baggage, when black people, in general, face oppressive threats or trauma in society, black individuals in organizations experience adverse intrapsychic outcomes, viz., negative emotions and rumination – impelling their social identities to become activated, along with their already activated organizational identities, typically leading to identity conflict or psychological interference.

Notions of feeling ‘less-than’ are passed on from one generation to the next, shaping the relationships that black people experience with the white ‘other’ (Alleyne, 2004). Alleyne (2004) refers to an ‘internal oppressor,’ which she describes as that part of the self that carries historical and *trans*-generational pain. Bartley (2013) recounts her experience with internalized oppression where she constantly second-guessed her competence because she – a black woman – believed that she was not as good as white people. Alleyne (2004) claims that the result of internalized oppression is low self-esteem and self-hate – neither of which is particularly helpful for leader emergence. Social stereotypes – experienced by black people – additionally contribute to them not putting themselves forward for leadership positions, because they believe that it is ultimately pointless (Lowe, 2013). White (1970) emphasized the need to develop a precise and practical theory of black psychology through the use of authentic black experiences.

## Methodology

This research followed an inductive approach, which is a process where qualitative data is used to build theory (Gioia et al., 2013). This approach was then layered onto the hermeneutic phenomenology research approach. A cross-sectional design was adopted for this research. Participants were chosen on the basis of having lived experiences (namely those of black professionals holding leadership positions) relevant to the focus of this research (Groenewald, 2004), as well as their willingness to share their experiences (Laverty, 2003).

The scope of the study focused only on black South African professionals within organizations in Gauteng, South Africa. This research adopted the definition of ‘black’ as provided by Ndinda and Okeke-Uzodike (2012), but for the purposes of this research, excluded Colored and Indian professionals and only focused on ‘African’ professionals as per this definition. Additionally, the study focused on employees that had a minimum of 5 years working experience and encompassed professionals in both leadership positions (8 out of 9 participants) as well as those not yet occupying positions of leadership (1 participant – P9), but who, with these credentials, possessed the sufficient requirements to move to and aspire to a leadership role.

‘Professional’ in the context of this study meant employees who had a minimum of one university level bachelor’s degree or equivalent. Employees who did not possess relevant qualifications were excluded from the study, even if occupying a leadership position. Since the researchers aimed to select atypical black South African professionals that were in sufficiently different industries/sectors from one another (to increase the possibility of unique organizational stories), but in the economic hub of South Africa – Gauteng Province – they chose a purposeful sample selection strategy (Ajjawi and Higgs, 2007; Maxwell, 2008). See Table 1 for a list of participants and their demographic data.

**TABLE 1:** Participant demographics.

Participant demographics									
Region	Participant no.	Identify as female	Identify as male	Age	Generation	Industry/sector	Years of working exp	Leadership role (Y/N)	Qualifications
Gauteng	P1	x		37	Millennial	Banking	18	Y	Master’s degree
Gauteng	P2	x		43	Gen X	Transport	20	Y	Bachelor’s degree
Gauteng	P3	x		34	Millennial	Financial services	10	Y	Master’s degree
Gauteng	P4	x		33	Millennial	Engineering and construction	11	Y	Master’s degree
Gauteng	P5	x		35	Millennial	Education	11	Y	Master’s degree
Gauteng	P6	x		37	Millennial	Real Estate	14	Y	Bachelor’s degree
Gauteng	P7	x		39	Millennial	Financial services	18	Y	Master’s degree
Gauteng	P8		x	44	Gen X	Finance/Banking	25	Y	Master’s degree
Gauteng	P9		x	45	Gen X	Government administration	15	N	Bachelor’s degree

Semi-structured, in-depth interviews were conducted with the participants (Ajjawi and Higgs, 2007). According to Laverty (2003), it is best to ask open questions with only a few direct questions included. The reason for this chosen approach was to allow the discussion to be led by the participants, thus allowing the interview process to remain as close and true to their lived experiences (participant voice) as opposed to getting ‘simulated’ accounts of what the participants thought that they had experienced (Laverty, 2003). All interviews were audio-recorded, with the permission of the participants (Whitehead, 2004). Field notes were also utilized to complement the data gathered (Groenewald, 2004).

The researchers decided to follow a hermeneutical research approach, which is similar to phenomenology, but does not require the researchers to ‘bracket’ or set aside their biases, but instead, the researchers utilized these biases in the interpretive process (Laverty, 2003). Thus, using the license to utilize bias, which is a fundamental part of the hermeneutic phenomenology approach, the researchers, who are both black South Africans and, thus, have insight into the lived experiences of black professionals and black people in South Africa, used these lenses in the interpretation of the results. Additionally, the reflexivity approach used during interviews acknowledges the fact that that the researchers are a part of the social world that they study, and it is a process of reflection on the role that subjectivity plays during the process of research (Palaganas et al., 2017). During this process, the researchers had to continuously reflect on their values as well as how their backgrounds, locations and assumptions may have affected their research practice (Palaganas et al., 2017). Reflexivity encouraged the relationship between the production of the interpretations and the challenging of those interpretations (Alvesson, 2003; Laverty, 2003). The reflexivity process was aided by the keeping of reflective journals to record all assumptions and to force the researchers into reflective attitudes (Laverty, 2003). Peer debriefing assisted with the robustness of the interpretations, to enhance trustworthiness of the research process (Torres and Hernandez, 2007).

**FIGURE 1:**
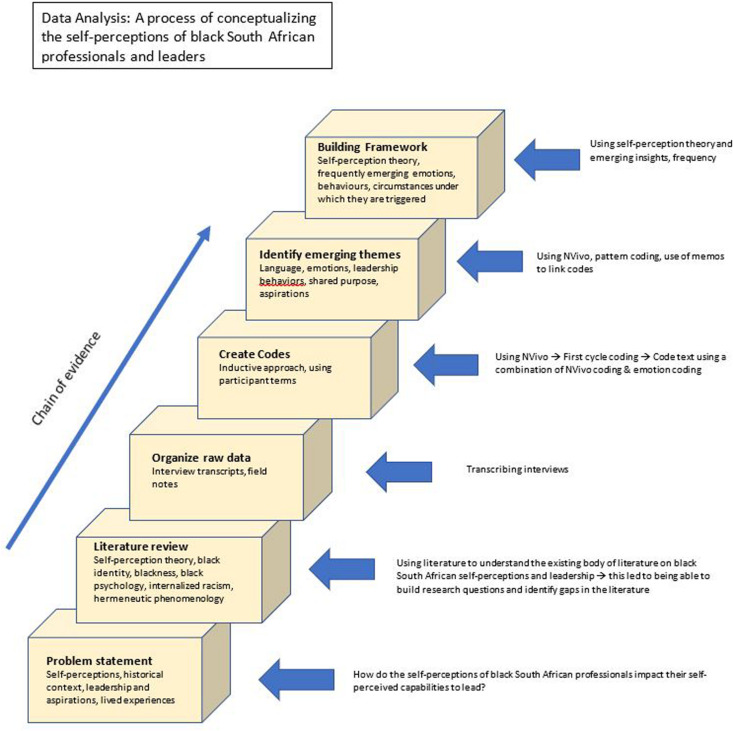
A demonstration of the data analysis approach employed in this research.

Thematic analysis was used to analyze the data, which was a method that allowed for first order constructs (the interpretations and constructs of the participants) to be identified and layered with second order constructs (the researchers interpretations, understandings, and constructs) (Ajjawi and Higgs, 2007; Gioia et al., 2013) – unearthing themes and patterns of living and/or behaviors that were identifiable (Aronson, 1995). The first order constructs, as much as possible, kept the terms used by the participants (Gioia et al., 2013). Using transcribed data from the interviews, the patterns of experiences could then be identified (Aronson, 1995). These patterns were derived from direct quotes or through paraphrasing of common ideas (Aronson, 1995) - see Figure 1 below for a depiction of the analysis process followed.

Coding was used to label and keep track of the interviews. In addition, the hermeneutic circle was utilized as a metaphor for interpretation and understanding (Ajjawi and Higgs, 2007).

Transferability in qualitative research serves the same purpose as that of external validity or generalization in quantitative research (Riege, 2003) – thus, specific coding and analysis procedures were utilized during the data analysis stage to assist in ensuring transferability (Riege, 2003). The use of reflexivity (Laverty, 2003; Groenewald, 2004; Ajjawi and Higgs, 2007), creating texts that were reliable to the experiences of the participants, which can be understood by insiders as well as outsiders, lack of dishonesty and consistency of research conclusions that reveal the complexity of the situation were critical in achieving adequacy in the hermeneutic approach (Laverty, 2003).

## Results and Discussion

### Self-Perceptions

Race is a huge part of how people view themselves and others, and therefore is an important consideration in leadership perception (Festekjian et al., 2014). Acton et al. (2019) propose that how individuals behave in their roles as either leaders or followers is largely influenced by how they view themselves as leaders within a given environment.

To gain insight into the self-perceptions, the research engaged participants on their lived experiences through self-reporting which allowed perspective into their thoughts, feelings and behaviors (Schwarz, 1999) in relation to the South African organizational and historical context. This approach allowed a journey of discussion into the lives of black professionals in and outside organizations, allowing us to build a view; albeit tentatively, of the emotions, attitudes, behaviors and identities that they exhibit in the different contexts, including the triggers (circumstances) thereof.

**FIGURE 2:**
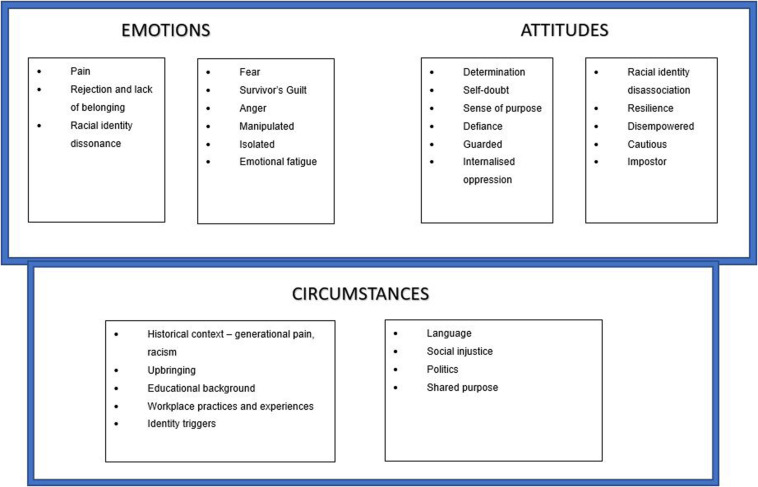
Self-perceptions of black professionals and leaders.

Our research found that the self-perceptions of black South African professionals and leaders could be diagrammatically represented as follows (Figure 2), a conceptualization of the emotions and behaviors of black professionals and leaders as well as the circumstances under which these are triggered that was systematically built from the participants’ own accounts of their lived experiences and the associated emotions and behaviors they exhibited, which were then mapped diagrammatically using self-perception theory.

These self-perceptions give insight into the behaviors and attitudes (shown in the attitudes ‘box’) that atypical leaders exhibit in leadership roles and the various, sometimes conflicting, emotions (shown in the emotions ‘box’) that are triggered as a result of the various circumstances (shown in the circumstances ‘box’) such as intergeneration trauma, racism in the workplace, and others shown in the diagram. We delve deeper in the rest of the paper to attempt to discuss some of these themes and how they present as either barriers or enablers in the emergence of atypical black leaders.

### Identity

#### Racial Identity and Leadership Capability

Although a few black professionals shared that their black identity was a disadvantage to how they were perceived in terms of capabilities, for instance, P8 purported: *“… [being black] takes away a lot. People see your color before they give the advantage, before you say anything. My white counterpart comes in no matter how little experience he has, but people already take him seriously upfront. Until you say something that resonates with them [white people], you will be taken as a token,”* a larger number of research participants argued that their identities contributed positively to their ability to lead. They argued that black identity allowed black leaders to relate better with their employees, the country’s majority population, adding that black leaders were more considerate, understanding and more relaxed in their leadership enactment. Additionally, black identity was considered a strength on the basis of shared history, and the very unique black struggles and experiences that were part of that history. P7 stated: *“We know how it is to work hard. We know how it is to not have. I feel that we should be able to operate in unstable environments*… *where we can take other people’s issues into consideration. If someone is having a problem*… *comes late to work because the taxi broke down. Even if you are driving a Mercedes Benz [these days], you know what it is like with taxis breaking down, because you have been in it.”*

#### Racial Identity Dissonance and Disassociation

Colonization’s biggest injury against the colonized is the imposed eradication of their identities, which has been deliberately and brutally replaced by large parts of the colonizers’ identities. Even more cruel was that the shedding of colonized individual’s identities were ‘sold’ as improvements to their civilization, not unlike a plastic surgeon who sells cosmetic surgery to those in desperate psychological health deficiency. Nkomo (2011) argues that colonization dehumanized and socialized Africans to despise themselves and everything that made them who they are, including their history and their culture, making them see themselves as uncivilized. In South Africa, specifically, Hocoy (1999) proposed that the study of black racial identity was crucial to determine the extent to which discrimination experienced by black South Africans may have damaged their mental health. Bartley (2013) suggested that black leaders, like herself, could occupy a leadership position in relation to the ‘other’ and be competent and effective, all the while holding the white leader experience in high esteem. Our research results revealed that black leaders could distance themselves from ‘other blacks’ when they were in leadership positions. They disassociated themselves from the constructs of their black identities, in attempts to detach themselves from the negative stereotypes that were linked to being black, for example, being lazy and maintaining facades of conformity. Several participants associated black leadership with perceptions of being a *‘better black.’ P2 remarked:* “… *There’s the best black syndrome: ‘I am better than other blacks. I am not like them. I am different. I don’t do the things that they do. I do not think like them. I am not lazy’.”* This disassociation is a form of psychological colonization, as described by McKay (2008), specifically the concepts of racial alienation and alienation from one’s culture, own language and history. In addition, Nkomo (2011) explains that the work of Fanon detailed the practice of colonialism that created a sense of self-alienation and a deep need for assimilation with the dominant group. As an example, some participants shared that they did not ‘see’ racism, nor held any strong political opinions – although this was a significant part of their lived experiences as black South Africans. P7 purported: *“*… *I never experienced racism when I was growing up. Maybe it was around me and I never saw it. Even when I went to a Model C school [former whites-only, semi-private schools – subsequently mixed schools since the end of formal Apartheid in 1994], I never experienced it.”* P7 goes further by claiming: *“I feel like I have graduated to not being able to see color. I do not see color here*…*”*. Showunmi et al. (2016) explain that early formative encounters with racism coupled with social class transitions allowed middle class blacks to utilize class membership as a tool to minimize the likelihood of experiences of racial discrimination.

A respondent shared that she tried to ‘fit in’ with her white friends, which often resulted in uncomfortable conversations. She divulged that her white friends interacted with her because they believed her to emulate characteristics more palatable to them (a more ‘civilized black’), and viewed her to be ‘less black’ than a ‘regular black’: *“*… *they say: ‘Oh, but you aren’t the same as other blacks’*… *that is the challenge. I am not the blackest black, and not the better black. I am just me*… *Sometimes I feel strongly about things that affect a lot of blacks, and maybe I am seen as black by the black people, or sometimes I see things differently and the white people may think she is not a hothead like the others*…*”* (P7). This example illustrates the fact that black professionals may sometimes struggle, finding themselves moving between racial identity dissonance to complete disassociation. Racial disassociation can be adopted as a coping mechanism, to escape being drawn into the bleakness of the black experience.

Other black professional respondents exhibited signs of racial identity dissonance. One shared that she did not relate to ‘black interests,’ largely due to her upbringing which had isolated her from the black lived experience as it pertains to South Africa. She struggled, for instance, to communicate in ethnic African languages: *“I think my upbringing and interests have me almost at a point where I have to pick a side. I struggle in terms of black leaders. I don’t fully relate to my black leaders. If we switch to ‘vernac[ular],’ I can only go so far. If we are talking about where I went during the weekends, I went hiking or I went to weird mushroom farming*… *the responses I get: ‘Oh! So, you are one of those Model C girls?’ So, it becomes harder then to build connection points with them [other blacks or black leaders] as a collective*…*”* (P1).

This is the context of some black South Africans, the select few in the emerging black middle class that experienced life in white neighborhoods and schools, resulting in the destruction of their black identities, including language, black relationships and the complete eradication of their black culture. These black South Africans form white relationships and, in some cases, these relationships lose their ‘innocence’ and connection when the reality of South Africa’s racial segregation infiltrates their consciousness – P1 explained: “… *I noticed race in the first year of varsity*… *where you might have been high school mates with white friends, but all of a sudden you are no longer mates. People pass you and you are like: ‘Hawu besiblomile [we were hanging out] over lunch 3 weeks ago, and now all of a sudden it is as if we have never seen each other’.”* Similarly, Bartley (2013) narrates a childhood story of a day when she walked hand-in-hand with her white friend who, upon seeing other white kids, quickly let go of her hand. To Bartley (2013) this was experienced as a surreptitious judgment of her blackness and, on a psychological level, made her feel inferior in relation to whiteness. Similarly, P1 was hurt and bewildered by the rejection which would continue into her adulthood. This experience made her feel alienated, as she realized that the fullness of her black identity would never be completely accepted by her white friends. In the workplace, she also realized she could not relate with her black colleagues either – she did not speak their language, did not use the same accent when conversing in English, nor did she share the same interests – because her upbringing had been deficient in these aspects of her black identity. This feeling of being in ‘limbo’, reminiscent of Du Bois (1897) ‘twoness’ or double-consciousness, can contribute to racial identity dissonance: *“*… *you can get to relate to both white and black leaders, but then it is hard for any of them to literally put you into their inner circle*… *because of the trust issues”* (P1).

Other experiences that were shared involved interactions with white colleagues, out of necessity, that contributed to black professionals feeling forcibly isolated, desperate to mingle with black colleagues, but prevented from doing so because they were generally placed in roles where they were the only black person in those groups, alienating them from other black people: *“I felt like a sell-out, to a certain extent, because then even though you wanted to mix with other black people, you also needed to sit with your fellow [white] trainees, and you ended up feeling like: ‘What the hell”’* (P5). According to Utley (2016), the quality of blackness is comprised of an awareness of the conditions that resulted from colonization through poverty, disease, wretchedness, and racism. For black people that may have been removed from this experience, it could contribute to their absolute inability to relate to what ‘black’ means in the context of South Africa, thus contributing to their disassociation and rejection by other black people. Even though some black professionals portrayed a lack of awareness of their blackness, through disassociation, our findings also showed that other black professionals were extremely proud and protective of their black identity and drew strength from their blackness. This research also found that many black professionals were becoming increasingly intimate with their blackness, and were indeed moving toward viewing themselves as *‘black, adequate and non-inferior’* (Hall et al., 1972, p. 4), echoing the pleas of Steve Biko in his evidence given at the SASO/BPC trial in 1976 (Gwaambuka, 2017).

### Historical Context

Nkomo (2011 p. 366) points out that literature has repeatedly painted Africa as a failed continent, labeled “*‘irremediably corrupt’; ‘hopeless’; ‘criminal’; ‘ungovernable’ or generally in ‘chaos,”* however, she argues that there is a tendency to ‘forget’ and to diminish Africa’s challenges to a deficiency in leadership and management when, in fact, these challenges can be fully attributed to the continent’s colonial past and its now post-colonial present. South Africa, specifically, has a history of double colonization by both the Dutch and the English (Nkomo, 2015). South Africa later experienced the establishment of the apartheid system in 1948, which resulted in complete domination of the African, Colored and Indian people (Nkomo, 2015). Despite being referred to as a postcolonial country – a term which is used to describe a nation regarded as being at the end of colonization but still experiencing the ongoing effects of colonialism on its present condition Nkomo (2015)-Hocoy (1999) points out that black people in South Africa still continue to experience racism and prejudice, even post-apartheid.

In South Africa, Nkomo (2015) noted that, from the time of colonization to the times of apartheid, the rule of the White minority firmly established a rejection of Africa and African agency.

#### Historical Context as a Barrier

##### Intergenerational trauma

Black professionals in South Africa carry the pain of their parents and grandparents as if it were their own – because intergenerational trauma is very real for them. Their children also will carry the same pain, hurt, angst and fear, as their own, and will need to be constantly reminded about how society treats them and how inferiority will be imposed on them, and so it shall continue. Apartheid laws might have changed, but the violence of race-based poverty and the belief system, and its accompanying assumptions, that black people are not only inferior, but are also less than fully human, persists. Alleyne (2004) suggested that black people carry perceptions of being ‘less than,’ that are passed down through generations. Our participants frequently referred to intergenerational pain as a result of experiences that were narrated to them, including how these historic events continue to affect them today: *“*… *whether it is how we were raised, whether you grew up without your dad in the house because he was a migrant worker or fearing Apartheid, it manifests. this memory of what happened in the 80’s and 90’s, of what your parents and friends have been telling you about people in exile [in opposition to Apartheid and living outside of South Africa]. And you have never really fought Apartheid, nor went into exile yourself, but there is this inbred anger*…*”* (P3). These narrated experiences, passed on from generation to generation, opened wounds that were triggered by the perpetual existence of oppressive practices in contemporary work and social environments. P3 further related: *“The stuff that is passed on through generations, and stuff that will be passed on for years to come, for some reason we don’t break that. We don’t seek healing, counseling and any of that. I am not part of the born-free generation [those born post-Apartheid and in democratic South Africa] but, as a 34-year-old, why did it bother me so much that they had voted for XX [a white political party in South Africa]?”*

In South Africa, 26 years after the establishment of democracy and the abolishment of Apartheid, black professionals still carry the memories of the past and persistent sentiments about slavery, with some respondents revealing that they associated black leadership with being a ‘house slave.’ It is all very much different, but still very much the same. This is due to the strong element of white control, particularly economic control, that they felt was exercised to keep black employees ‘in their place’ when occupying these roles. P5 asserted: *“*… *it is those people who are the house slaves. The ones who get invited to dinners, because they can keep you in your place.”*. Furthermore, the vividness with which the respondents remembered their own first encounters with Apartheid and their experiences with racism is a testimony that their lived experiences still hold enormous power over their lives today. Most respondents shared that their earliest memories of racism were from childhood, when South Africa was still in the depths of Apartheid: “*I remember the ‘no blacks allowed’ signs everywhere, when we were growing up”* (P2). P6 recounted: *“I remember this one time when our parents forgot to bring along our birth certificates, so we were not allowed to go into Kokstad. So, my parents had to leave us with the Police*… *the first time I felt the segregation. We were not allowed to go into this town because we were black kids”*; similarly, P5 remembered: *“I heard him say: ‘Bloody kaffir’ [derogatory term, akin to ‘nigger’ in the United States]*… *I never even told anyone. I was in shock*… *He bana! [exclamation of shock]*…”.

P1 shared a telling memory, explaining that, as a child, she always wanted to sleep over at her white childhood friend’s house in the white suburbs. It was closer to their school and when sleeping over there, got three more hours sleep as compared to when she would sleep at her own house in the township: *“*… *I used to say: ‘I like sleeping at Bronwyn’s house. I can wake up at 7 am’*… *it is a small thing, but that drove my current behavior of always wanting to live next to where I work versus waking up at half-past-five, and traveling to get to school*…*”*. Acutely aware of the race-based spatial development of Apartheid, she innocently did not fully grasp how her definition of success was being formed through the white lens – experience and affluence. Achieving this goal, would become the ultimate validation that she had ‘arrived’ and become one of ‘them.’ Fanon explains this phenomenon: “… *for the black man, there is only one destiny. And it is white”* (Bartley, 2013, p. 170). As is evident in this story, the little black girl grew up normalizing that the majority of black people struggle for the good things in life, i.e., living in an under-resourced township, without a family car, lack of sleep due to spatial planning (keeping black people at enormous distances from city centers and where companies/work were situated), braving the cold in badly constructed dwellings and in public transport queues, having to commute to work in crowded public busses and 15-seater taxis, whereas the white experience was radically different, with very short drives or walks to schools and places of work – with coffee shops along the way, living in well-constructed homes in leafy suburbs, and even having time to meet up after work because of the proximity to homes. Sadly, the beliefs that black people are resilient, strong and built for struggle, are ingrained in the minds of society and social systems that were created to propagate it as normal. Because of this, black people are raised believing that the only way to achieve success and workplace credibility is to continuously desire to be white or becoming something close to white (to forego their black identity) – go to white schools, speak their language and in their accents, live in their suburbs, become their friends, learn and play their sports, have hair that closely resembles theirs, marry their sons and daughters, and eventually gain their approval. P5 reflects on this: *“Whether it is in the way that you were brought up in your home, society’s expectations, or the way in which the media brainwashes you, blacks are always on the receiving end. We are the ones who are always being colonized.”*

##### The workplace as a trigger

Workplace circumstances that fuel the behaviors and self-perceptions of our respondents were found to be similar to the workplace oppression findings of Alleyne (2004). Alleyne (2004, p. 4) shared the types of oppression experienced by black people in the workplace, including:

• Having their presence ignored.
• Lack of eye contact from white colleagues when it mattered.
• Frequent incidents of being excluded.
• Constantly being described as aggressive, scary, angry, frightening, threatening, a problem, difficult.

Our research uncovered similar findings, the details of which have been shared extensively in other sections of this paper. Our research also found that workplaces can be triggers for black professionals, seemingly embodying Apartheid, racist contexts, demeaning attitudes and microaggressive behavior in present form. P3 claimed: *“… we have all these societal issues that have taken place, and we are among the first or second generation who are in corporate. I don’t know who lied to us and said that we are going to be coming into these spaces and that they will welcome us, and that they were going to be representative of who we are. And then we enter here and realize that this is apartheid all over again.”* (P3). She further reflected on white people’s demeanor toward her at her workplace: *“It is something I have experienced in this bank over and over again. These white people look at me and they see Elizabeth [maid], who washes their underwear in their home. The only difference between Elizabeth and I is that I was appointed because of affirmative action. Otherwise, I’d still be Elizabeth. The difference between Elizabeth and I is that I have a couple of degrees and I speak better English*… *but they do not view you as being their equal. They don’t even view you as someone that can engage them in any meaningful way: ‘Why are you here? You should be at home doing what black women do*… *and when you challenge them, they don’t like that as well because now they are forced to engage you.”* She explained that some of the things that triggered and enraged her ranged from political issues to the persistence of the master-slave relationships of the past, and to being forced to work with older, white employees. Some white people believe that black people have been given unfair advantages through employment equity policies, and they are oblivious to any privileges that being born white have given them – and so seek out political alliances to ensure the sustainability of such privilege and to challenge the new order in the country. In her view, they perpetuated the existence of the racial problems in the country through their affiliation to oppressive political parties: “… *I got in on Monday and I am sure ‘yibona laba abavotele iFF Plus’ [they are the ones who voted for the FF Plus – right-wing, white Afrikaans party]* … *that is why you have to be radical in the workplace, because they never stop fighting. Automatically I just elevated a nothing into a massive argument for nothing. It bothered me because I felt powerless in that moment. I felt: ‘How dare they?’ These people, we have given them an opportunity to live and work amongst us. In fact, they must pack their bags today and leave.”* (P3). Another female respondent (P7) expressed feelings of anger and explained how she had chosen to address it in her everyday work life: *“*… *I have progressed from being ignorant to being angry, to take white people on at every opportunity I would get because I was so angry. To say: ‘What can I do in my space that can make a difference?’ I applied it to my overall life, so that I don’t go home and sleep with a heavy heart. So, whenever I do my job, I do it well.”*. P8 expressed anguish at the fact that, regardless of how good he was, his work environments kept reducing him down to merely a black skin: *“*… *regardless of the experience I come with, I still have to prove myself*… *part of it is because of the color that I bear. Part of it is in my name*… *I get evaluated first on the color of my skin versus what I have on the table, what I deliver. A lot of people draw conclusions.”*.

##### Black leaders and authority

Bartley (2013) speaks at length about her experience when she believed that, as a black woman, she was not as good as white people. One of our respondents suggested that black people were conditioned not to question white people, and that this contributes to the difficulty to challenge white authority in the workplace. It is important to highlight that 6 out of the 9 respondents highlighted that owning and portraying authority was not their natural inclination. P5 explained: *“With a white colleague, it just makes it so difficult. They could literally come and tell you that the sky is green*… *because of the way that we have been trained to view them”*. Cranmer and Harris (2015) confirm that, indeed, the decisions of white leaders are unlikely to be exposed to racial prejudice. Our research found that for many black leaders, authority felt unnatural because of negative leader expectations, under-representation of blacks in organizational leadership and persistent negative consequences of speaking back to white people/people in authority during Apartheid and colonization. Black people were made to believe that their rightful place was in a position of subservience and recognizing the value of being more closely aligned with those with power – what Biko termed a multifaceted situation of oppression (Hill, 2015). Moreover, this could be attributable to why some black managers were perceived to be more lenient on white employees: *“*… *[Black leaders] do not develop other blacks. They tend to be hard[er] on the black employees than they are on the white employees”* (P2).

##### Government failure

Respondents shared that they were cognizant of, and angry at, the failures of the government to ensure complete transformation, evident through the persistent under-representation of black professionals in strategic roles, while senior white males continued to hold on to senior positions and economic power in organizations. *“In a state-owned entity, you’d think we’d be spared from some things*… *but white management stay here until they die. You have this thinning layer of middle-aged white men, who are fifty and above, and I am cognisant of South Africa’s 27% unemployment rate. And I can tell you now that the 90-odd percent of unemployed graduates are blacks. These are [white] people who started under Apartheid, so they’ve got special privileges, bonuses, and benefits. They were working for a good 10 years under Apartheid before black people could work here”* (P3). Furthermore, black professionals believed that their experiences of marginalization in the workplace, which continued unabated, were encouraged by government failures and corruption, which served to perpetuate white stereotypes about black leadership: “*When our black government leaders mess up, it reflects badly on us*… *it fuels their views about us*…” (P1). P8 stated: *“It goes back to whether our government is willing to enforce policies that it has come up with. They have nice policies, but they are not enforcing them. These things perpetuate exclusion for us at work. If you force [white] people to put a plan in place, they will. That will be our plan, but they will not train you, they will not skill you.”*

#### Historical Context as an Enabler

##### Challenging historical practices

Kuada (2010) suggests that leadership in Africa is shaped by historic events. South Africa’s dark history of pain, unique to black South Africans, has had a very lasting impression and its effects are still visible and penetrate all the spheres of black lives, including their professional lives and definitely the leadership behavior of black leaders.

##### Collective and shared purpose

Our research found that black professionals feel group pressure and have a deep need to bring change to the black community, as well as to positively influence the circumstances of other black professionals in the workplace: *“If I fail, it does not just represent me*… *it’s going to be a representation of all black people”* (P4). Black professionals, like many marginalized groups, believe themselves to be representatives of a whole, and thus believe other black professionals to be reflections of themselves. There is a shared purpose, an unspoken understanding amongst black professionals that stems from shared trauma, cemented with an unspoken pact to revolutionize the fate of black people. The responsibility to bring change in the lives of those previously underprivileged in their communities and in the workplace, and ultimately to bring change in the country, lies at the doorstep of every successful black leader: *“*…*I feel a certain responsibility and my job is finding businesses that create certain job opportunities for [black] people who do not have access to what I have had access to. When I see the black leadership here, they have the same purpose as I have here. I feel strongly that we should have more South Africans who are working in these environments versus having whites, because there is the understanding of the history and goal of where we want to ultimately take the country”* (P7). Our respondents have all claimed that they have, more recently, become unapologetic about vocalizing issues relating to transformation. They assert that there is an emotional fulfillment that comes with achieving this change. Sometimes this expectation and shared purpose can feel burdensome, they claimed, but it was one that our respondents were committed to. Some of our respondents have vocalized that, as black professionals who have ‘made it,’ they felt a sense of ‘survivor guilt’ for ‘leaving others behind.’ The guilt has further propelled them to help others. P3 shared: *“*… *the personal fulfillment that I am making a difference. Knowing that little girls can go to school, instead of fetching firewood and water, because we have put a pumping station there*… *she can study and when she’s done, she can switch off the lights. Then I can say that I have done a great job on this earth.”*

##### Management across color lines

Our black leader respondents informed us that they enforced change through deliberate and empathetic leadership toward black employees – they purposefully give black employees deserved platforms previously withheld from them. Wyatt and Silvester (2015) claim that black employees can benefit from black managers who previously navigated the labyrinth, have the networks and can create the opportunities. Roberts et al. (2019) reiterated that creating opportunities for black people to bring their authentic selves to work boosts engagement and helps employees contribute more to the organization. Interestingly, most of our female leader respondents revealed that their leadership behavior varied across race, with most indicating that they were more supportive and understanding of black employees but less patient and tougher on white employees, mostly attributed to strong feelings about the history of the country: *“I am more lenient toward black women. They are allowed to make mistakes. They are allowed to grow. They are allowed to be late. A black woman can tell me now: ‘Ukuthi uAunti azange afike’ [that the helper/maid did not come in] and I’ll get it. A black woman will say: ‘I didn’t look [did not prepare] and I’ll pass her my things’*… *white people wouldn’t even dare”* (P3). P7 similarly stressed: *“I may have a softer spot for younger black people. It may not come naturally for me to go to a white male of the same age and do that. I think it is a race thing. You look like me, you sound like me, I want to nurture you.”*

### Unconventional Pathways for Atypical Leader Emergence

#### Enabling Pathways

##### Upbringing

Leader emergence can be partly attributed to upbringing. Our research highlighted two areas as being big contributors to the determination to succeed in our sample group. The first was early parental enforcement of the importance of education, which some respondents highlighted as particularly important while raised during a time of Apartheid conflict in South Africa and which could have easily been a distraction, resulting in very different outcomes. P7 reflected: *“… they [parents] were quite conservative when they were bringing us up. They never allowed to us be affected by the noise. They stayed away from politics, even on TV. The TV would be switched off when there was too much volatility being shown. So, I lived in my head. I read a lot of books. I was removed from the South African context.”* P8 claimed: *“*… *my mum always drilled in us that hard work pays off, never to take the easiest way out, and to pay our dues. We had to sit at the table, with our books open, and that has always stuck with me throughout my life.”*. The second was the fact that the majority of black professionals experienced financial difficulties at home, which translated into tertiary affordability problems, and therefore they had to pay their own way through tertiary education – often by working multiple jobs in order to afford fees, boarding and meals: *“I come from a family of 12. My parents have never paid a cent toward my senior education. Everything I did was because I hustled. I tried to get scholarships*…*”* (P8).

The Apartheid policies and funding disparities in white and black schools ensured overcrowded classrooms, working class instruction with low and under-qualified teachers, little to no educational resources and materials, and therefore contrasting access to higher education. Additionally, and by law, there was no financial aid and banks did not give out loans to blacks during Apartheid (Ocampo, 2004). Post-Apartheid, even with the democratic government’s attempts to equalize education, educational inequalities persisted because of the inseparable nature of race and socio-economic status. A handful of ‘lucky’ black students were recipients of bursaries or government funding. Generations of black parents remained low-skilled and either had no jobs or could only get lowly jobs from which no financial security could be gained. This is the dark history and present reality of most black families.

Some black students, who are now black leaders and professionals, were part of the ‘missing middle,’ a phenomenon where one was not eligible for government funding because, according to qualifying criteria, they are deemed ‘too well-off’ for financial assistance: *“… I was one of those you know*… *called the missing middle*… *considered too rich for a bursary and too poor for a student loan*…*”* (P5). This is a huge challenge in South Africa, as the criteria for funding does not reach all those that need it, mostly black students. The financial challenges that confront many black families contribute to the arduous path traveled by black professionals in their journey to become actively employed and eventually into leadership. Those who do make it ‘through the net’ carry a huge responsibility to back-fund the educational opportunities of siblings, family and other community members, while also financially required to support families (known as ‘black tax’). The political and social systems still fail to undo the economic injustices of the past that continue to haunt black South Africans today. The ‘Fees Must Fall’ movement of 2015, which saw black students revolt against financial exclusion from universities, particularly for the inclusion of financially disadvantaged black students, was a result of the hopelessness and anger that accompanies the ongoing injustices. Thus, for these black professionals recounting their own journeys to reach where they are is important (for others to hear), as well as being inspiring.

##### Leveraging white relationships

Acton et al. (2019) divulge that the leadership emergence process is part of a greater context that involves both informal relationships and formal structures within the organization. Our research revealed that, in some instances, black professionals quite deliberately leveraged white relationships to get ahead. This practice was not malicious in its intent, but was calculated and sometimes, a desperate one. P1 divulged that all of the advancements in her career had been linked to white leaders with whom she had premeditated relationships: *“A lot of my career opportunities up until this current one have been given to me by white leaders. I have always constantly leveraged white people to grow.”* She also revealed that once the relationship had achieved the intended outcome, she swiftly moved on, careful to ensure that she did not linger too long, lest she be labeled a token. In some cases, however, the relationship can grow into a long-lasting mentorship, even after it has delivered the necessary returns: *“*… *my biggest growth came from a manager who was white*. *He is the person that opened the doors for me. He is the person who taught me how to behave in corporate. He is the person that taught me not to react emotionally. Whether it is in corporate or personally, he has always been in my life”* (P8). It is evident that black professionals are still mindful of the differential power dynamics in the work environment and have found ways to navigate this otherwise very oppressive and discouraging reality in their favor. However, the ever-present fear of being labeled an employment equity appointment is still very real, and these relationships, although useful, can overshadow the capabilities of the black professional. It is a precarious balancing act, thus making it hard for these relationships to be genuine and long lasting. It is also important to note that the white counterparts in these relationships are usually older, professionally senior and carry influence in the organization. The white leader in these relationships is more likely to be a willing participant if the power lies with him, and if he does not consider the black professional a real threat to his own current and future position in the respective company.

#### Pathways as Barriers

##### Language

On the 16th June 1976, black students in South Africa were killed and jailed by the masses across the country as they fought against the use of Afrikaans language as the medium of instruction in schools. Afrikaans was the language of the oppressive Apartheid government, and white-minority ruling government. Decades and generations after this historic demonstration, and many black lives later, the use of Afrikaans as a tool to exclude, demean and dominate continues in present day South Africa. The use of Afrikaans – one of South Africa’s 11 official languages today – as a form of exclusion in educational institutions and in workplaces has negatively affected the experiences of black professionals, forcing them to find coping mechanisms in order to survive and rise. Moreover, it is important to highlight that, as a result of the dark history and the lives that were lost in the fight against the use of the language, Afrikaans represents a particularly severe psychological and emotional trauma for black South Africans. Participant P8 emotionally narrated how, as a black man from the township [under-resourced and under-developed, racially segregated urban area] who did not speak any Afrikaans, he had to find a way to succeed in a tertiary institution where he was instructed in Afrikaans. He emphasized how this experience left him with the realization that he had to learn to survive in a world that is hostile towards him: “*When I talk about resilience, it also touches on marginalization*… *your ability to deal with a situation where people want to exclude you, and you need to find your way to the table. Some of us grew up with marginalization from almost birth. When I was doing my engineering degree, when it was still called Universiteit van Teknologie [University of Technology], I did my maths, technology and my digital technology in Afrikaans. I will never forget that. My books were in English and my class medium was in Afrikaans. From those types of experiences, you learn to have a coping mechanism. You learn to have a thick skin. You learn that the world we live in will never give you anything on a platter. You will take these things through life with you*…*”*. These experiences have built resentment and opened up wounds inflicted by the country’s colonial past, by entrenching the same practices in the supposedly more inclusive educational institutions and workspaces. The use of Afrikaans in the workplace, contributes to the feelings of inferiority, self-doubt, isolation and many other negative outcomes for black people: “*In a meeting it happens where people will speak in Afrikaans, and then it is almost like they are deliberately leaving me out of the conversation*… *it is a cold war”* (P5). On occasion, where black professionals would retaliate, by responding to written Afrikaans communications in an ethnic African language, it would lead to a stand-off.

Post-Apartheid, South Africa adopted 11 official languages in an attempt to give back some dignity and their identity to black South Africans. However, in white, Afrikaans-dominated workplaces, black professionals remain disempowered and subjected to the language of their colonizers: *“*… *one of the first things they did was to start speaking Afrikaans in meetings. I’m sure that they spoke Afrikaans in meetings before I arrived. There were black people there who didn’t speak a word of Afrikaans and they didn’t speak up for themselves”* (P3). The workplace struggle with language for black professionals is expansive and, often, black employees that are subjected to this treatment do not stand up for themselves. However, to minimize exclusion from pertinent work discussions and decisions, and to stay connected to networks of power, there are instances where black professionals make an effort to learn and speak Afrikaans. It is, however, evident that younger black professionals are rebelling against this abhorrent practice of deliberate marginalization, but it remains yet another battle, another hoop to jump through that professionals from other groups do not need to contend with.

##### Permission seeking

Important to note is the power dynamics in these white relationships that black professionals leverage. Black professionals felt quite threatened to do the sponsors bidding, with the fear that if they did not, they would lose the benefits of sponsorship. Respondents shared that they had to be careful to ask for ‘permission’ from the white sponsor before considering involvement in other projects, career moves and seizing workplace opportunities: *“… I asked my current boss, and said: ‘Those people are looking for me, should I go?”’* (P1), while others stated that they were careful not to ruffle any feathers, lest the sponsor decided to soil or even sabotage their future career prospects and networks, if they became ‘too big for their boots’: *“*… *you literally have to sit and watch what you say. Long term, you have aspirations, but you think: ‘I am going to need these people”’* (P5).

### Workplace Experiences

Black professionals were exposed to extraordinary and unforgiving circumstances in the workplace – they had to fight to claim legitimacy, a seat at the table, credibility, and equality.

#### Workplace: Barriers

##### Isolation

Most black professionals have, at some point in their career, been ‘the only black’ or ‘the first black’ within their organizations, especially in positions of leadership: *“The difficult thing in the space in which I work is that there are very few people of color, which means half the time I am surrounded by either white people or Indians*… *which makes it very difficult to build those relationships within the environments in which I work*…*”* (P8). Black professionals alluded to the fact that they sometimes turned down roles and struggled in spaces where they would be ‘the only black,’ even if it ultimately affected or delayed their career growth: *“They offered me the job and then I told them ‘no’*… *because I was going to be the only black. I don’t want to be the only black. Do I even want to be in this place [where] nobody looks like me? I cannot even identify with anyone. Then [organization name] gave me a better offer. I get there on day one, and then I realize I am going to be the only black again. I want to be able to speak my own language in the workspace. I want to be able to identify with black people. Then you go to the work environment and it is like, whatever you learnt at varsity about self-identify, you have to undo*… *in order to get to certain levels on the ladder”* (P5). Samdanis and Özbilgin (2020) assert that diversity in the workplace remains a much-desired social undertaking and that atypicality in the leaders of an organization’s composition can be used as a measure of the level of diversity and democracy of an organization. Yet, in a supposedly diverse context of South African organizations, black professionals questioned how it was possible that in a country that was made up of a majority of black people (79% of the population), that they would still be a minority within the management and leadership of organizations: *“*… *sometimes you sit and think: ‘Is it possible that in an organization this big, I am still the only black that sits in this senior management panel?”’* (P6). This and similar questions led to doubts in our respondent sample about whether they were appointed on merit and for their capabilities.

##### Exclusionary practices

Black professionals detailed their experiences of exclusion in the workplace, through the actions of white employees/managers, as well as those practices that were embedded into work policies: *“*… *it is the little things*… *people setting up meetings at 7h00am and you start coming to work at 6h45am*… *they can’t leave you out. People start creating a migration process where you work in two places, where half of Exco is in one building and you are the one left in the other building, and no-one says anything. Things like weekend dinners [that you are not invited to]*… *where people take workplace decisions at those dinners or craft certain things*… *you get to work on Monday, there is this long email that these things need to be delivered by Friday and nobody’s told you the context. So, you need to find those coping mechanisms.”* (P8). Policies that were meant to be equally beneficial for all workplace employees were, in fact, found to be subtly oppressive to black employees due to the unspoken differences in expectations of white and black professionals. P5 reflected: *“*… *as a black female parent, I have a daughter. I have to pick her up. While you have a white colleague in the same position, same everything, and who also leaves at 12. However, for me it is viewed as general laziness because I leave at 12* … *I find myself still worried about clocking in and clocking out, and I find myself still worried about being watched and monitored*… *put under a microscope. As a black female, you are ostracized. You are regarded as ‘that group’ who always complains.”* This participant alludes to the topic largely viewed as the giant elephant in the room, privilege. Showunmi et al. (2016) suggest that the role, that the privileged and those in positions of power play in creating policies, prevents the full inclusion of the minorities in society (or organizations). In this instance, black females within organizations are still the minority in those spaces.

##### Racism

During Apartheid, race, militarized police, and government bureaucracy were the three main devices used to inflict violence against other human beings who were not white. In post-Apartheid South Africa, black South African professionals continue to be subjected to blatant and subtle racism in the workplace, despite the establishment of democracy more than two-and-a-half decades ago: *“*… *as a black woman, I was placed in branch banking. The Jewish white boys were at equities, at head office, even though we were in the same pay grade. None of the white guys and white women were in branch banking. I lived in Sandton [affluent suburb] at the time. The rationale of putting me in a branch close to Soweto [a black township] was so that I could be ‘close to home,’ because the assumption was that, as a black woman, I come from a township*… *but the white kids have been accommodated in the Sandton offices, because they all live in Sandton. They would never have thought to sending all those white kids to a branch close to Soweto.”* (P3). Many white colleagues avoid talking about race, for fear of sounding or being seen as prejudiced, and so resort instead to strategic color-blindness (Roberts and Washington, 2020) or non-racial rhetoric as ways to continue to support white privilege (Milazzo, 2015). Their silence, complicity and material benefit during Apartheid, together with their current inaction for substantive reparations, are not easy realities for them to absorb or own. As a result, white fragility was raised by our respondents when discussing the inability of mainly white colleagues to venture into difficult conversations about differences and asymmetrical workplace experiences – typical reactions that were shared were defensiveness, physical or psychological withdrawal as a result of an inability to engage in constructive engagement, and talking about the periphery/extremes and in the language of groups when describing black behavior and action as popularized through the media. Other participants described blatant racist incidents with either their colleagues, managers or clients. P2 recounted: *“*… *I did have a lot of Afrikaner clients. They would call me ‘meisie’ [girl]*… *you know, using such derogatory words”*. P6 shared: *“*… *I had a tenant*… *when I was with another organization. They told me straight that they were not willing to talk to me. They wanted to talk to a white person.”* Racism can be attributed to causing psychiatric disorders such as anxiety and depression, low self-esteem, academic degradation and generally low life satisfaction (Pierre and Mahalik, 2005). Some of our respondents shared personal stories of some, or all, of those disorders.

##### Rejection

According to our black professional respondents, the workplace was an environment that was not accepting of their kind: *“*… *it becomes very important that, through the journey, you have the ability to continue to take punches, stand up, come back and deal with the same people who probably do not want you in those spaces.”*. The workplace was described as a battlefield, and required immense mental and emotional fortitude to wake up every day, prepare to go into the office and face the battle over and over again: *“I need to be sharp and on the ball all of the time. I must be a fighter every day. Every day you’ve got your guard up.”* (P3). P5 described boardroom rejection and ridicule, in similar ways in which other respondents told us that sometimes it was overt and other times in the form of microaggressive behavior: *“We must have been in a boardroom somewhere. The person was giving a presentation and the person did not look at me, not once. He looked at everyone else and made eye contact, but not once did that person look at me. He was a white male*… *he was blatantly ignoring me.”*. Showunmi et al. (2016, p. 14) describe microaggression as *‘brief and commonplace daily verbal, behavioral, or environmental indignities, whether intentional or unintentional, that communicate hostile, derogatory, or negative, slights, and insults.’*

A qualified professional, female respondent P2 explained that her inputs were not accepted as credible, unless a white colleague verified its validity: *“… I remember when I was quoting all this analysis. I drew on stats in terms of why I was proposing a certain stance in pricing this particular transaction. One lady asked a question, and I answered. She then turned to another colleague of mine who wasn’t the same race as me to confirm with him if I was right*… *right in front of me*…*”*. P5 elaborated on the psychological and behavioral effects: *“*… *in an environment that is Afrikaans male-dominated, they have certain ideas about black people. So, you have to censor yourself. You get home, and it is not that you might have had a hectic day, but you are exhausted. Everything that you are thinking and feeling you must analyze. How is this going to look? By saying this, is it going to be labeled as angry? If I do not say anything, am I going to be falling into the typical: ‘Oh, they do not say much.”’* Our respondents shared stories of having to bite their tongues in meetings, swallowing rage, holding back their tears, and choosing not to walk out of meetings and companies even though every ounce of their being was screaming out for them to do so.

#### Workplace: Enablers

##### Coping through black support networks

The lack of representation hinders the ability of black professionals to build relationships amongst each other, and signals that not all types of identities are congruent with professional careers. When black professionals enter work environments, they can experience feelings of being overwhelmed and alone. Thus, they resort to seeking relationships with professionals that ‘look like them’ outside of the workplace and with whom they can share their experiences, the precarity of their positions, and about the alien workspace dynamics which they have to navigate: *“I have got a network of black professionals outside of work that are doing similar things at similar levels. We share knowledge and share challenges. We also talk about how to build resilience in terms of certain situations”* (P8). One of the respondents shared that she had joined a ‘blacks only’ social club that was formed by black professionals in her organization. She noted that, after having bounced from employer to employer and feeling isolated because she was *‘*the only black*’* in those environments, the ‘blacks only’ group had been a great source of comfort. She added that this support structure had contributed to her prolonged stint in her current organization, versus other organizations where she lacked similar support: *“… it is a formalized group which is only us – black people in the department that belong to this group. That gave me some hope that there are some people who realize that this is a problem. It is a social group where we get together, maybe once a term or something, where we unwind*… *it is the first environment where I have had that*… *and maybe there are other factors that influenced it, but this is one job that I have been at the longest”* (P5). P5 went on to state: *“*… *I just had to find people who looked like me, who shared the same experiences*… *but I could not even articulate to them that is the reason why I wanted to associate with them.”*

### Aspiration

#### Aspiration as a Barrier

##### Self-sanction

According to Festekjian et al. (2014), projective identification can influence the aspirations of the affected party and, in some cases, cause them to completely bury their own aspirations and force them to not want more out of their careers in their current organizations. Our research determined that, for our sample group, black South African professionals were well aware of the perceptions held by white people regarding their workplace capabilities. Lowe (2013) assert that black professionals have internalized these beliefs and end up believing them to be true, as claimed by P8: *“There is almost no expectation. We are there to make up the numbers. We are there almost like kids. We are there to be seen and not heard.”(P8)*. The research revealed that some black professionals believed that their success was attributable to the fact that there were not enough black people to compete for the roles they occupied, rather than the fact that they deserved their roles based on merit or because they were exceptional: *“…a friend of mine said that I’m not special. The only reason that things are happening so quickly is because there’s a lack of choice of people. So, if there were four or five of xx’s, it would have been a lot harder for me to have made it” (P1)*.

##### Self-deselection

A number of respondents deliberately did not put themselves forward for senior or leadership roles in their organizations. This was due to a combination of reasons, including the fear of being perceived as over-ambitious, the fear of being stigmatized as tokens, the fear of being promoted out of turn, of not getting support or respect from white employees, self-doubt and the fear of having to deal with racial challenges in the workplace. P1 noted: *“I have had a lot of people come up to me and say: ‘Why don’t you step up?’ Perception-wise, it is going to look untidy. I haven’t even proven myself in this role, but I’m already wanting this [higher] role because it is an executive role. So, I have said ‘no’ to that*… *I’m pulling myself back, but I’m doing it rationally.”* When offered career advancement opportunities, some black professionals revealed that they had, in some cases, turned down the opportunities out of fear that they would suffer irreparable emotional damage as a result of the experiences that they would encounter: *“*… *This is a position that has to be black and this is a position that has been offered to me. It worries me. I do not want to put myself in a position where I have to work twice as hard in order to prove myself”* (P5). P5 further maintained: *“I refuse, not because I don’t think I’m capable, it is because*… *you will be dismissed, you will be ignored. It requires of you to keep building yourself, because somebody else keeps cutting away at your self-esteem. I am afraid that I might not rise to the occasion.”*

##### Diminished leadership aspirations

Some black professionals shared that they did not have career aspirations within the organizations at which they worked, e.g., P8 remarked: *“I do not have leadership aspirations here.”* Our research found that even though some black professionals may not have had leadership aspirations, they occasionally felt *obligated* to take up leadership roles because there were not enough black people in the organizations, consequently placing them in positions where they would be *expected* to step up: *“I do not have grand ideas and aspirations of leadership, however, I think because of the position that I find myself in right now*… *yes, there are not a lot of us*… *I might find myself in a position where [I am] forced to kinda do it*…*”* (P5). She believed such roles would be offered to her because the organization needed to achieve transformation/quota figures, and not because she was perceived as competent. For some black professionals, this knowledge had already contributed to their diminished aspirations within their organizations. The research further noted the witnessing of being directed into certain workplace disciplines, and other negative experiences of other black leaders in leadership positions, also contributed to the reluctance by black professionals to pursue career advancement, e.g., P8 shared: *“*… *if you are going to find a black Exco member, they are either responsible for one or two things: HR and Risk. You can go look at it*… *but when it comes to entities that generate revenue, that are critical, they don’t play a role.”*

#### Aspiration as an Enabler

##### Aspiration and racial identity

Our research revealed that black professionals wanted to see leaders that looked like them within their organizations – such representation of black leaders was seen as motivational impetus, which allowed them to visualize their own career prospects within their organizations. P1 stated: *“I look in the levels above me and see if there are any black individuals or black leaders and, if I do see black leaders, it gives me hope that there are chances for me to grow. It is important to look up to leaders that look like you, who have succeeded with the challenges that you are now experiencing. I promise you that this, kind of, also related with job movements any time that I got to a job*…*”.*

### Intersectional Themes

#### The Multiple Shades of Black and Career Progression in ‘Post-colonial’ Organizations – Additional Barriers to Atypical Black Leadership Emergence

Intersectionality describes the relationships amongst various aspects and modalities of social interactions and subject developments (McCall, 2005). It is the ‘*idea that social identities such as race, class, and gender interact to form qualitatively different meanings and experiences*’ (Showunmi et al., 2016, p.3). Showunmi et al. (2016) explain that ethnic and gender identities contribute significantly to the self-concept, however, they are often incorrectly considered independently and in isolation of other identities, which limits the understanding of the fullness of an individual’s experiences. It has become common that in some instances, intersectionality be used as substitute for oppression, without providing the details of what specifically is intersecting, or how (Carastathis, 2018). Showunmi et al. (2016) explains that intersectional perspectives combine feminist and multiculturalist views to better understand the nuances, specifically, of women’s experiences.

Our findings highlighted the discrimination that exists in organizations toward darker skinned black professionals, placing further barriers in atypical leader emergence. Lighter skinned black professionals face slightly fewer barriers in their leadership journey as they are regarded as more ‘acceptable’ in organizations and, therefore, receive better career and wage opportunities (Goldsmith et al., 2007). This form of multi-layered discrimination is harsher against black female professionals. Darker black female professionals, who also have bigger physical frames, are additionally penalized. Bartley (2013) shared a narrative about how small she felt every time her white peers questioned the nappy texture of her hair, black hands and white palms – and she eventually discovered a way to hide her ‘black experience’ in front of white peers. Black women have experienced heavy societal discrimination over their appearance, with society promoting ‘whiteness’ – slender build, long straight hair – as ideal. In pursuit of this white perfection and scaling hierarchical access, black women in South Africa have, for a long time, hidden their natural, curly hair, and consequently their identity, behind long straight ‘weaves’ made from hair from white and Asian women and delved in skin whitening creams: *“*… *light skinned black women are afforded different, and even better, opportunities. Thin black women are given other opportunities than those who are short, dark and chubby*… *there are additional hurdles. You are black, then you are [a] woman; then you are dark; then you this, then you that*…*we fight”* (P3); “… *the less good-looking, darker slaves were considered less eloquent, and so their jobs were out in the field. The ones that simulated and had characteristics closer to [whites] made them more appealing to the masters. When you are lighter skinned, you seem smarter by their standards*… *you are ‘the yes baas [boss]’ kind”* (P5). Intersectionality also brings focus to the subject of whiteness, which due to its privilege is often not addressed in organizational diversity literature and discourse (Showunmi et al., 2016). Kalish and Luria (2016) argue that individuals emerge as leaders only when they possess attributes that are similar to those of the perceiver or more specifically, if they fit the ‘prototype’ and possess the attributes that the observer (or perceiver) associated with leadership. In this case, the ‘prototype’ would be that a black female leader must have ‘long, straight hair, be of slim build, light skinned, and speak English fluently with a ‘white’ accent,’ thus, as close to a white female as possible. In contrast to Bartley (2013), who felt compelled to ‘hide’ her black features, the findings show that black South African leaders are rebelling against this oppression of their identity with P3, a black female leader, strongly asserting and imposing her physical appearance in the workplace, a phenomenon that is picking up momentum in black society recently. Black people have begun to proudly, and vocally, defend their identities and heritage. Black female professionals are proudly and unapologetically embracing their natural, thick and nappy locks more, as expressed by P6: *“*… *I deliberately want to have big hair, because I know it offends people. Black hair is not tidy, and people want you to conform in a number of different ways*…*”*. Black people have regained their voice and speak out loudly against multinationals that attempt to demean black racial identity, e.g., Clicks (leading pharmacy, health and beauty retailer) being the latest to experience the full might of black rage after posting a TRESemme advert insulting black hair in favor of white hair. After nationwide protests, the company was forced to take the offending brand off their shelves and to apologize to the nation.

Equally, blacks from urban backgrounds tangibly get more workplace opportunities, and in-company promotions, than those from rural backgrounds.

Language, more specifically, the ‘black accent’ is another ‘shade’ that may impact the career progression of black professionals. English is not a first language for the majority of South African black people, instead, their first languages are indigenous languages such as isiZulu, isiXhosa, sePedi, depending on tribal background. The majority of black professionals attended schools where the medium of instruction was an indigenous language. With the changes in the country, especially post-1994, access to Model C [mixed, formally white, better resourced] schools increased for black communities, especially the more affluent families: *“… you have got your model C private school blacks*… *it is easier for you to take that leadership position, and be assertive, [if you attended a model C school]”* (P5). The Model C schools’ medium of instruction is in English and they boast a complementary racial mix. Therefore, black learners who attended these schools gained the ability to speak English fluently while also being exposed to learners and cultures of different races. However, a larger number of black South Africans could only attend schools in the under-resourced, black-only townships, limiting their exposure to spoken English, at least at pre-tertiary level. Our research found that professionals that were less fluent in English, were at an additional disadvantage and were more likely to be side-lined for upward opportunities in organizations. This is largely due to the perception that ability to eloquently communicate in English is directly linked to intelligence, an unconscious bias that has also infiltrated black communities’ views that ‘better blacks’ are closer to being white (superiority): *“*… *God forbid you bring someone with a strong African accent. It is the house slave versus the field slave*… *the ones that will sit on boards, the ones that will be in management, that are brought in by the white superior colleagues, because they can relate to them*… *you are more relatable than someone who went to a typical township school.”* (P5).

**FIGURE 3:**
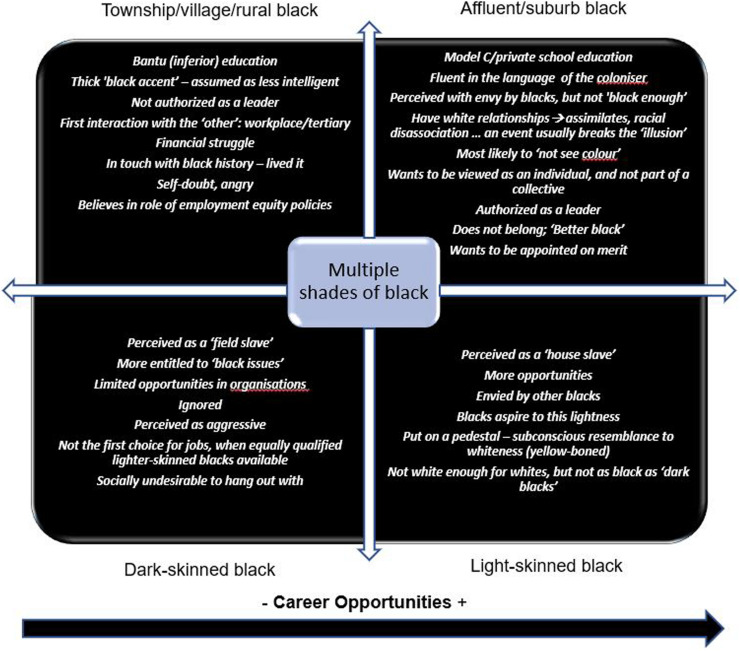
Classification of black professionals.

The above diagram (Figure 3) attempts to graphically represent the quadrants in which black professionals are classified by unconscious bias, creating additional hoops and barriers to overcome toward leadership emergence. These shades often overlap. At any point in time, an individual is a mix of two, sometimes even three shades that can be brought on by a change in circumstances perhaps, as an example, social class transition (Showunmi et al., 2016), such as a relocation from the township to the suburb. However, an individual always has one dominant shade, and this is the one that others may ‘see’ at face value and on which an individual is judged more strongly – this is often the shade that will influence their career progression. There are other secondary shades that are not represented, e.g., physique.

#### Consequences of Atypical Black Leadership in the South African Context

##### Representation

Our findings revealed that black professionals and leaders find themselves extremely racially isolated within organizations. This circumstance leads to black professionals turning down roles to avoid being placed in positions where they will be ‘only black.’ This means that organizations also feel the negative impact of this lack of representation by potentially losing talent – through an inability to attract or to retain black professionals who simply refuse to be part of organizations that do not have representation. Secondly, the research found that leadership aspirations amongst black professionals could be linked to racial identity representation in the leadership structures of their organization. Thus, we can conclude that black professionals who feel represented in the leadership in their organizations are more likely to aspire and work toward leadership positions in the organization because they believe it is possible for them to attain. This could be utilized as a retention and transformation strategy for organizations. Black professionals and leaders shared that due to isolation, lack of support and lack of leaders that understand and relate to challenges that are specific to black people, they often seek support outside the organizations in the form of black networks. The consequence of having black atypical leaders in an organization is that they provide the much needed emotional and structural support for black professionals and, more specifically, provide empathetic leadership that can relate and mentor other black professionals in a way that white leaders simply cannot. Again, representation can therefore provide emotional support, create loyalty and give guidance and a sense of belonging to black employees in an organization contributing to an overall improved workplace experience for black professionals.

##### Policy making

Organizations that are untransformed and lack diversity are prone to practicing policies that discriminate and exclude as the policies themselves are created by the dominant group. Therefore the policies are more likely to protect the privilege of the dominant group, with little to no consideration about the inclusiveness of such policies for the marginalized group. The consequence the presence of atypical leaders is that policies can be created with the marginalized in mind, thus reducing levels of discrimination.

##### Management bias

Black South African leaders shared that they exhibit deliberate color bias in their leadership enactment. The color bias stems largely from negative feelings, such as resentment and pain as a result of South Africa’s painful history as well as the leaders’ need to balance the scales by providing black employees an increased likelihood to succeed, an opportunity they would not otherwise receive from a white leader. There is a general feeling by black leaders that the system was historically created to favor white employees, to support them and therefore they do not ‘struggle’ to make it. The consequence therefore of increasing the number of black atypical leaders is that this phenomenon might eventually become diluted with time, due to improved diversity and a general feeling amongst black leaders that organizations are transforming, are representative of the country’s demographics, and reducing the need to take matters into their own hands through intentional acts of bias.

##### Ubuntu

Ubuntu, a South African, black cultural philosophy, has been cited as one that could be a competitive advantage (Nkomo, 2011). It is a philosophy that black people in South African are raised under, and one that is deeply entrenched in their attitudes. Thus, black atypical leaders would, because of their cultural background and their upbringing, possess the spirit of Ubuntu, and organizations who intentionally attract black leaders in their organizations would reap the benefits of this competitive advantage. Ubuntu could contribute toward wellness, loyalty and inclusiveness in organizations, benefits that can be realized through the leadership of atypical black leaders.

## Conclusion

This research has added to the body of knowledge by clearly mapping the paths taken by atypical black South African leaders to occupy leadership positions within organizations, detailing the barriers and enablers that contribute to the culmination of this endeavor. Furthermore, this research has shed light into the self-perceptions of black atypical leaders that shape their emotions and behaviors in leadership roles, an area that has been rarely covered in leadership research, but nevertheless an area of research that has never mattered more in a country that is deemed to be post-colonial, free of Apartheid, and a full blown democracy. The research is even more relevant to organizations that are committed to transformation and diversity in the wake of the country’s shameful history of exclusion. More than ever, it is imperative that black leaders are heard, as they begin to surface and tell their own stories, and in their own words.

The findings have shown that the paths taken to occupy leadership positions are long, arduous and atypical for black professionals. Upbringing clearly plays a big role in the chosen paths of aspiring black leaders. The early enforcement of education in times of turmoil contributed to the determination to succeed. The presence of parental figures who were successful and hardworking contributed to the motivation to do well. Financial struggle, e.g., the inability to afford education in their formative years, is a big part of the journey of black professionals.

Black professionals are highly conscious of the history of the country, and they carry deep-rooted memories of their childhood, Apartheid experiences, and the experiences of generations before them. The generational trauma is persistent and passed on through narrated stories and experiences of marginalization and violence against black people. The pain continues to be triggered in modern day experiences – socially, in organizations and all the way through to boardrooms. Furthermore, black leaders believe that the government’s policies have failed to deliver the transformation required within organizations. The history of the country, their workplace experiences, and shared trauma, all contributed to a collective purpose for black professionals – to bring change by pulling each other up.

Afrikaans as a language of oppression in educational institutions, and in the workplace, remains a large contributor to the difficulties faced by black professionals, creating additional hurdles for them to achieve and to function effectively in the workplace. Afrikaans carries a dark association for black people and being expected to ‘deal with it’ does nothing for the psychological healing of the black community and the so-called future, demographically representative organizational leaders of South Africa.

Furthermore, black professionals are required to work in environments that are still modeled to resemble the Apartheid era, in environments that reject them, isolate them from their own, and judges them considerably more harshly than their white counterparts. These experiences have led to feelings of pain, anger, self-doubt, fear, and guilt, while fortunately increasing their determination and sense of purpose. In these work environments, the message is clear – if you fail, you carry the burden of knowing that you have effectively blocked access for other black professionals seeking to walk the same path.

In order to climb the corporate ladder, black professionals sometimes form transactional relationships with white counterparts. These relationships do not fully serve them – but, in order to gain career advancement, they have to ‘seek permission’ from their white sponsors, stay just long enough to benefit, and be smart enough to know when it is time to move on for fear of being labeled a token. These relationships can be oppressive as black professional are encouraged to mind their tongue, keep their heads down, or risk being cast aside or sabotaged by the white ‘sponsor’, should they fail to toe the line. In extremely rare cases, these relationships can eventually become genuine, meaningful and even turn into friendship.

Racial isolation can contribute negatively to the emergence of black leaders, with black professionals choosing to turn down opportunities where they would be ‘the only black.’ Black professionals seek a sense of belonging and a sense of community with their own people in organizations. In order to cope with the onslaught of exclusionary and demeaning work experiences, they seek comfort within black networks. Within organizations, these networks, that are safe spaces for them, remain covert in their operation in order to protect the beneficiaries from victimization.

Black professionals are deliberate about the leadership positions they pursue, and the visibility of black leaders within organizational structures are an important motivational impetus for the aspiration of other black professionals, which is strongly linked to racial identity.

Black professionals are furtively judged on ‘multiple shades’ of their identities in regard to career advancement, and ultimately on whether they are ‘authorized’ as leaders. Being lighter skinned allows access to better opportunities as they are deemed ‘better’ and ‘less black.’ Indigenous school English creates the perception of incompetence, whilst Model C/private school English makes one sound ‘whiter’ and thus, a ‘better black.’ These seemingly minor aspects of identity all contribute toward determining the ultimate success and emergence of a leader. This holds true for blacks who had rural upbringings (a disadvantage) versus those who had urban upbringings (advantage).

This research has shown that, although black leaders are penalized for their blackness, they also perceive their ‘blackness’ as a strength. They believe that their ‘blackness’ contributes to their ability to lead, due to their unique history of struggle and accompanying resilience – a history which, in South Africa, is peculiar to black people. Steve Biko associated the term ‘black’ with independence and self-reliance, and the research has established that in order to emerge as a black leader in South Africa, where odds are heavily stacked against black professionals, a black professional has to be exactly that.

## Recommendations

Inter-generational pain is a very real phenomenon that has wreaked havoc on the psychological well-being of black professionals in South Africa. It is important that black professionals and black leaders are made conscious of their condition so that they may actively seek healing through therapy, as well as sharing and telling the stories of their lived experiences to people who look like them and who relate to these experiences. Actively seeking healing will assist black professionals to deal with their feelings of inadequacy, pain, anger and affirm their identity. Organizations must boldly acknowledge the painful history linked to the Afrikaans language and decisively enforce corrective and restorative reparations to protect and restore the dignity and psychological well-being of black employees. Although organizations must be inclusive in their approach, they must put more effort into eradicating the use of the oppressive, Apartheid-era microaggressions in boardrooms and in formal work engagements – this attack on the black psyche must be swiftly eradicated through the formulation of appropriate organizational policies. Organizations must provide a safe environment, without censor, for the creation and/or existence of harmless ‘black only’ networks and events that celebrate, promote and bring pride to the black identity. Transforming the demographic landscape within organizations, to be more representative of the country’s demographics, could contribute toward retention of black talent. Black professionals migrate toward the environments that will allow them to build relationships with other black professionals, create safe spaces where they can share experiences with people that look like them, can advise them, and are able to relate to their lived experiences. Black leaders have a sense of purpose, to positively impact black communities and black employees that fall victim to harsh work environments. Therefore, it is important that black leaders are provided with opportunities to mentor young and disenfranchised black employees, in order to fulfill this purpose. This is a very specific need that cannot be fulfilled by white people, because they cannot fully relate to the very unique black experiences that only black people share amongst themselves. Organizations can benefit from these interactions too, as they can retain confident talent that is truly authentic and engaged in the organization.

## Data Availability Statement

The raw data supporting the conclusions of this article will be made available by the authors, without undue reservation.

## Ethics Statement

The studies involving human participants were reviewed and approved by the Ethics Committee, Faculty of Commerce, University of Cape Town. The participants provided their written informed consent to participate in this study.

## Author Contributions

KA and AM developed the research ideas and methodology together. AM did the data collection and the coding of the data. The analysis and write-up were jointly completed. Both authors contributed to the article and approved the submitted version.

## Conflict of Interest

The authors declare that the research was conducted in the absence of any commercial or financial relationships that could be construed as a potential conflict of interest.
